# Dataset on potential large scale production of biosurfactant using *Bacillus* sp.

**DOI:** 10.1016/j.dib.2017.05.037

**Published:** 2017-05-25

**Authors:** Hesty Heryani, Meilana Dharma Putra

**Affiliations:** a Department of Agro-industrial Technology, Faculty of Agriculture, Lambung Mangkurat University, Banjarbaru, Kalimantan Selatan 70714, Indonesia; b Department of Chemical Engineering, Faculty of Engineering, Lambung Mangkurat University, Banjarbaru, Kalimantan Selatan 70714, Indonesia

**Keywords:** Biosurfactant, *Bacillus* sp., Kinetic model, Emulsifiers, Glucose, Surface tension

## Abstract

Surfactants are very important in industry. The cost of commercial surfactant production is still high and the surfactant demand is constantly increasing. Microbial production of surfactant known as biosurfactant shows commercial potency. Utilization of *Bacillus* sp. strain on glucose fermentation for biosurfactant production was then studied. This type of microbe was isolated from soil contaminated with palm oil. The selection of the strain was based on its ability to form emulsifying zone around the colony and its capability to grow compared with those for commercial bacteria of *Bacillus pumilus* JCM 2508. The results showed a potentially promising strain with high biosurfactant yields and low surface tension. For further scale-up development, the microbe performance in a fermentor was compared with those in a flask and a proposed model to predict the kinetic profiles of cell mass, biosurfactant and surface tension were also described. The data presented here are related to the research article entitled “Kinetic study and modeling of biosurfactant production using *Bacillus* sp.” (Heryani and Putra, 2017) [1].

## **Specifications Table**

**Table:** 

Subject area	*Biotechnology and chemistry*
More specific subject area	*Production of biosurfactant on glucose using Bacillus sp.*
Type of data	*Table, text file, figure*
How data was acquired	*The microbe of Bacillus sp. was isolated from soil contaminated with palm oil from a local area.*
	*The isolation was based on the Morikawa method*
	*The selection of the strain was compared to Bacillus pumilus JCM 2508*
	*The performance of the strain was evaluated on glucose fermentation*
Data format	*Raw, analyzed*
Experimental factors	*The type of bacteria isolation was based on the Morikawa method*
	*The strain of Bacillus sp. grew on inoculum media*
Experimental features	*Isolation of sample to obtain the strain was conducted. With a fermentation process, new strain of Bacillus sp. grew on glucose and produced biosurfactant. The effect of carbon to nitrogen ratio was evaluated. A new model was proposed to predict the kinetic profiles of cell mass, biosurfactant and surface tension.*
Data source location	*Soil sample was collected from the land contaminated with CPO (Crude Palm Oil) at CPO industry in Banten, West Java.*
Data accessibility	*Data were obtained from the experiment and accessible within this article*

## **Value of the data**

● The high demand of biosurfactant could be supplied by a fermentation process by employing a strain of *Bacillus sp*.
● The optimum condition of carbon to nitrogen ratio was evaluated.
● For further process and industrial application, the modified Gompertz kinetic model, for the first time, was utilized to predict biosurfactant and cell. Furthermore, a new modification to the modified Gompertz kinetic model was proposed to enable the excellent prediction of surface tension.
● The promising results in the fermentation process using 0.25 L flask were further evaluated in the larger volume of substrate media by using 2 L fermentor.
● The sequence alignment of *Bacillus* sp. BMN 14 was provided.

## 1 Data

**Fig. 1:**
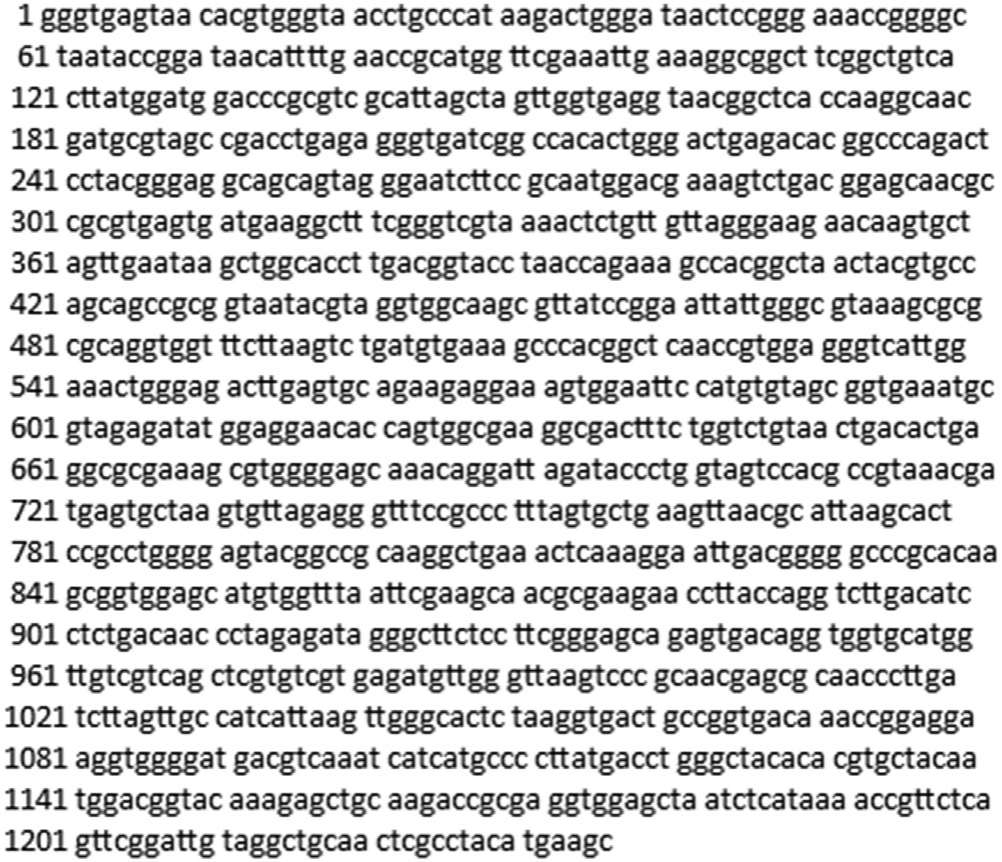
The sequence alignment of *Bacillus* sp. BMN 14.

The data presented here are not published in the previous work [1]. Fig. 1 shows the sequence alignment of *Bacillus* sp. BMN 14. The identification of the microbe based on the DNA sequence (barcode) using BLAST.

**Fig. 2:**
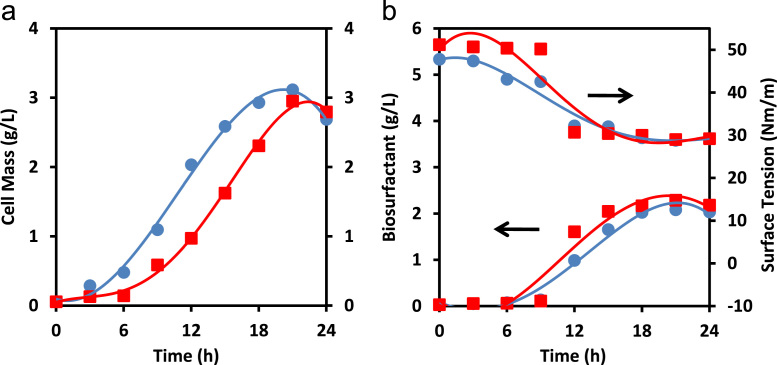
Profiles of: (a) cell mass; (b) biosurfactant and surface tension at initial glucose concentration of 1 g/L in 0.25 L flask (circles) and 2 L fermentor (squares).

Fig. 2a and b shows performance of *Bacillus* sp. BMN 14 on initial glucose fermentation of 10 g/L in 0.25 L flask and 2 L fermentor. Furthermore, the results in small volume (0.25 L flask) of media in the previous work were compared to those in a larger volume of substrate media by using 2 L fermentor. The profiles of cell mass, biosurfactant and surface tension showed similar performance of the strain in the two fermentors. It was also evident from Table 1 that all parameters of fermentation, -i.e., cell mass yield, specific growth, biosurfactant productivity and biosurfactant yield- showed quite identical values. Hence, this finding manifests the potential of applying this microbe in biosurfactant production in a larger scale or industrial application.

**Table 1:** Performance of *Bacillus* sp. BMN 14 in the comparison of flask and fermentor.

**Type of fermentation process**	**Cell massyield**^a^	**Specific growth**^a^	**Biosurfactantproductivity**^a^	**Biosurfactantyield**^a^
	**(g/g)**	**(h**^**-1**^**)**	**(g L**^**-1**^ **h**^**-1**^**)**	**(w/w)**
0.1 L Flask	0.766	0.191	0.098	0.514
2 L Fermentor	0.847	0.189	0.107	0.660

a Calculated at maximum cell mass concentration and biosurfactant production.

## 2 Experimental design, materials and methods

### 2.1 Microorganism and inoculum media

**Fig. 3:**
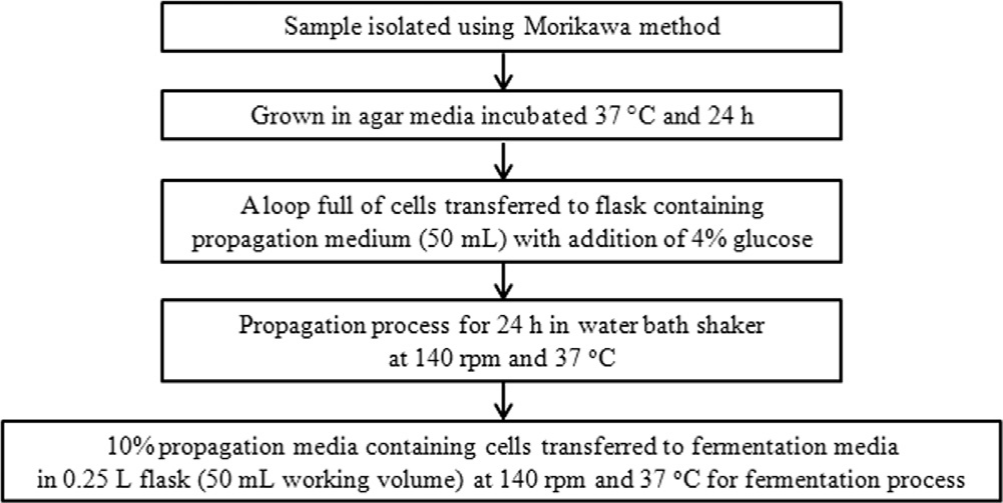
A typical process for microbial isolation and propagation.

The microorganisms, *Bacillus* sp. BMN 14 and BMN 27, were isolated based on the Morikawa method [2]. The isolation process and the sample were described in detail there [1]. The propagation process to grow the cell is illustrated in Fig. 3. It is clear that the growth of the bacteria took place in agar media, propagation media and then was evaluated in fermentation media for its performance [3]. The composition of propagation media was shown in Table 2.

**Table 2:** Composition of propagation and substrate media.

**Media composition**	**NH**_**4**_**NO**_**3**_	**KH**_**2**_**PO**_**4**_	**Na**_**2**_**HPO**_**4**_	**MgSO**_**4**_	**CaCl**_**2**_	**FeSO**_**4**_
	**(M)**	**(M)**	**(M)**	**(M)**	**(M)**	**(M)**
**Concentration**	0.04, 0.05 or 0.06	0.03	0.04	8.0×10^−4^	7.0×10^−6^	4.0×10^−6^

### 2.2 Fermentation process and design

**Fig. 4:**
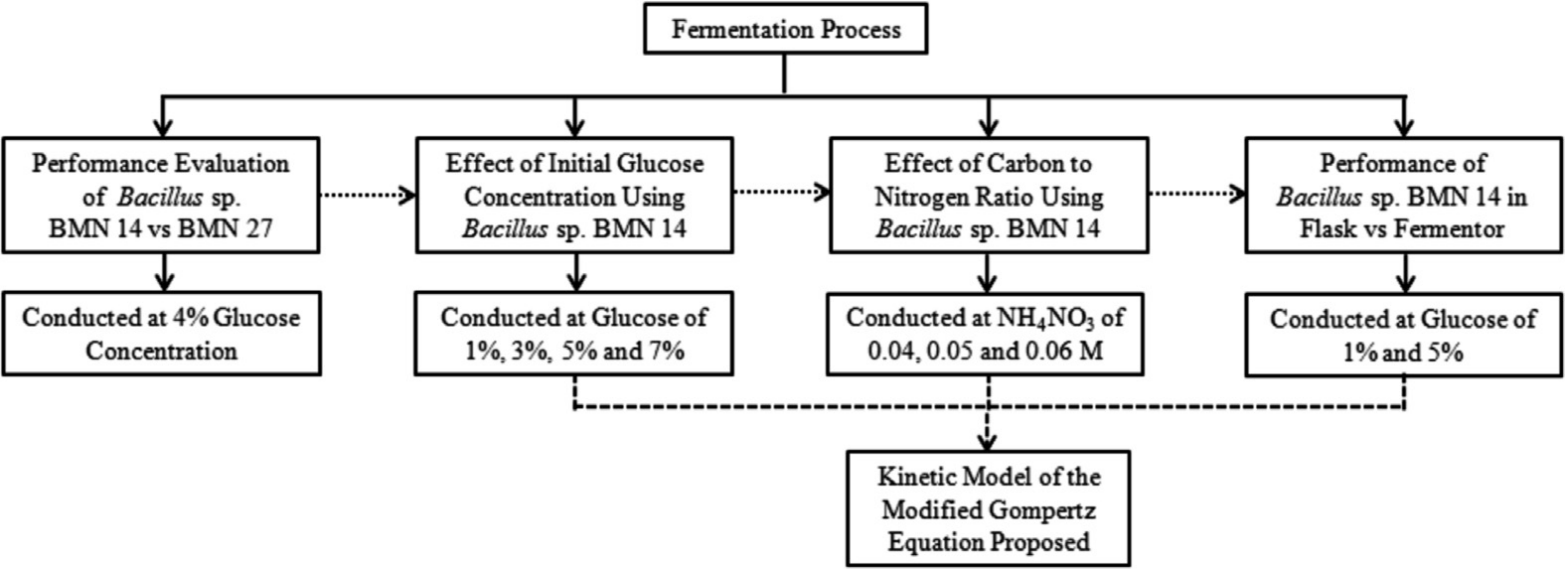
Design of fermentation process to evaluate the optimum condition.

The design of fermentation process is outlined in Fig. 4. The first step of evaluation was to determine the better performance between two types of microbes, i.e. *Bacillus* sp. BMN 14 and BMN 27. The selected microbe (BMN 14) was further tested to explore the effect of initial glucose fermentation (1%, 3%, 5% and 7%). The next step was to evaluate the effect of carbon to nitrogen by varying NH_4_NO_3_ concentrations i.e., 0.04, 0.05 and 0.06 M leading to ratio of carbon to nitrogen of 17.51, 12.36 and 10.56, respectively. For scale-up development and industrial application, the larger scale fermentor (2 L) was compared to the 0.25 L flask. Moreover, kinetic model of the modified Gompertz equation was also proposed to predict profiles of cell mass and biosurfactant production.

### 2.3 Samples analysis

**Fig. 5:**
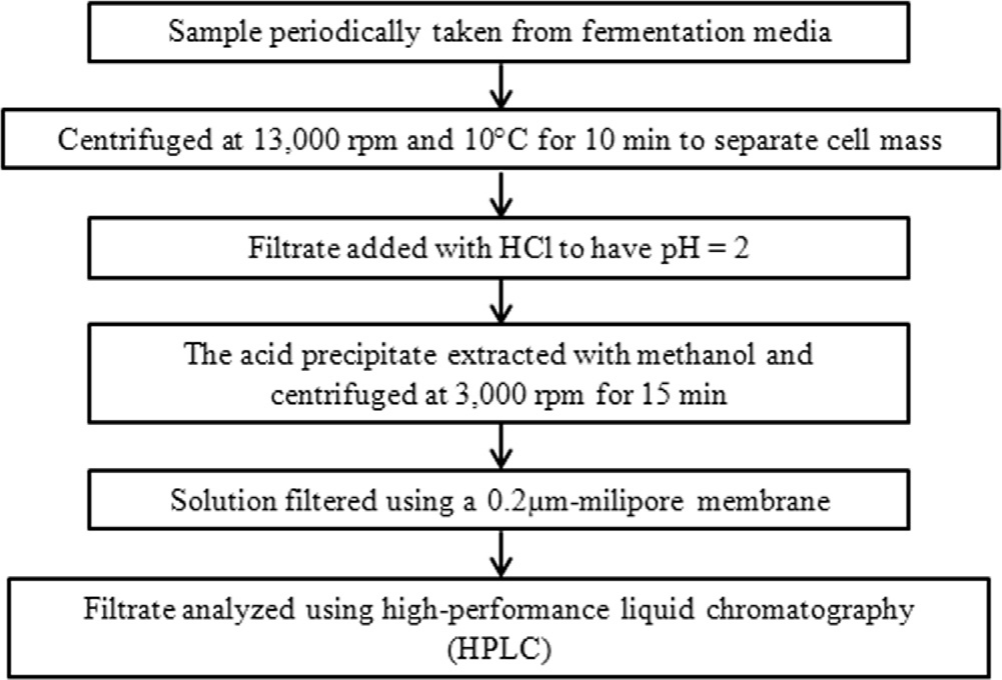
Diagram for biosurfactant analysis.

Analysis was performed to determine cell mass concentration, biosurfactant production and surface tension. The description of samples analysis was shown in Fig. 5. The cell mass concentration was evaluated using a dry weight method [1]. The filtrate of sample was analyzed to identify the biosurfactant concentration using high-performance liquid chromatography (Model Shimadzu HPLC 10A VP, Shimadzu, Kyoto, Japan) equipped with an RI detector and column (C18 Shimadzu, Kyoto, Japan). The column was maintained at 40 °C and acetonitrile solution with 1% acetic acid was used as a mobile phase at a flow rate of 1.5 ml/min. The calibration using pure biosurfactant purchased from Sigma-Aldrich was firstly conducted. The retention time for this pure biosurfactant was detected around 8.8, 9.6, 12.0, 13.0, 16.2 and 16.6 min. The observed peaks have similar trend as presented in the literature [4]. The surface tension was quantified by using Tensiometer (Model 70545, CSC Scientific Co. Inc., Fairfax, VA, USA).

### 2.4 Kinetic model

The kinetic model was proposed to predict profiles of cell mass, biosurfactant and surface tension. A solver called “fminsearch” in Matlab was used to estimate the parameters. The parameters are initially guessed in this code of Matlab. The objective function, i.e. [(experimental data−calculated data)/(experimental data)] based on mean squared error (MSE) was evaluated. If MSE achieved a convergence value of <1×10^−3^, the evaluation was finished. Otherwise, the procedure was repeated.
