# Supplementary material for: Dataset on potential large scale production of biosurfactant using Bacillus sp
Source: Data Brief. 2017 May 25;13:196–201. doi: 10.1016/j.dib.2017.05.037 (PMC5458641; doi:10.1016/j.dib.2017.05.037)
Supplement: Supplementary file 1 — Supplementary material [file mmc1.pdf]

Ref: DIB-D-17-00255, Title: "Dataset on potential large scale production of biosurfactant using *Bacillus* sp."

<u>To Whom It May Concern</u>

We declare that we have no conflict of interest for the manuscript entitled “Dataset on potential large scale production of biosurfactant using *Bacillus* sp.”.

Best Regards,

M.D. Putra, Corresponding author, on behalf of the coauthor

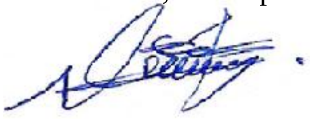

Meilana Dharma Putra, Ph.D.

Chemical Engineering Study Program

Lambung Mangkurat University

Banjarmasin 70123, Indonesia

Mobile: +6282281226215

Email: mdputra@unlam.ac.id;

Webpage: http://ft.unlam.ac.id/id/2015/08/27/mdputra/
